# Inclisiran: Efficacy in Real World—Systematic Review and Meta-Analysis

**DOI:** 10.3390/jcm14124163

**Published:** 2025-06-12

**Authors:** Álvaro Rodrigo Alaíz, Luis Corral Gudino, Leopoldo Pérez de la Isla, Héctor García Pardo, David González Calle, José Pablo Miramontes-González

**Affiliations:** 1 School of Medicine, Valladolid University, 47003 Valladolid, Spain; alvaro.rodrigo.alaiz@estudiantes.uva.es (Á.R.A.); lcorral@uva.es (L.C.G.); 2 Internal Medicine Unit, Río Hortega University Hospital, 47003 Valladolid, Spain; 3 Cardiology Unit, San Carlos University, 28040 Madrid, Spain; leopisla@hotmail.com; 4 Cardiology Unit, Río Hortega University Hospital, 47012 Valladolid, Spain; hgarciap@saludcastillayleon.es; 5 Cardiology Unit, Salamanca University Hospital, 37007 Salamanca, Spain; davidgcalle@saludcastillayleon.es

**Keywords:** inclisiran, c-LDL, real-world evidence, PCSK9, meta-analysis, lipid-lowering therapy

## Abstract

**Background:** Inclisiran is a novel lipid-lowering agent targeting PCSK9 via small interfering RNA (siRNA) technology. While clinical trials such as the ORION studies have demonstrated significant reductions in low-density lipoprotein cholesterol (c-LDL), real-world data (RWD) often differ due to variations in patient populations and clinical practices. **Methods:** This systematic review and meta-analysis adhered to PRISMA guidelines. A comprehensive search was conducted in MEDLINE for real-world studies evaluating inclisiran’s efficacy in reducing c-LDL. Articles meeting predefined inclusion criteria were assessed for quality and bias using RTI Item Bank. Data transformations were applied to harmonize median and IQR values to means and standard deviations for meta-analytic synthesis using RevMan 5.4. **Results:** A total of 3774 articles were identified, of which 7 studies comprising 1454 patients met the inclusion criteria. The meta-analysis revealed an average c-LDL reduction of 42.77% (95% CI: 37.42–48.12%). The subgroup analysis indicated greater reductions in patients receiving inclisiran alongside statins (45.67%; 95% CI: 36.64–54.71%) compared to monotherapy (37.53%; 95% CI: 29.91–45.15%). Discrepancies with clinical trials (e.g., 52% reduction in ORION studies) were attributed to baseline c-LDL differences and real-world adherence. **Conclusions:** Inclisiran demonstrates robust efficacy in real-world settings, achieving significant c-LDL reductions with a convenient dosing schedule. However, the observed discrepancies with clinical trials highlight the need for further RWD studies to bridge gaps in effectiveness and optimize therapeutic outcomes.

## 1. Introduction

Atherosclerotic cardiovascular disease (ASCVD) is one of the leading causes of mortality and morbidity worldwide [1]. One of the defining characteristics of ASCVD is the progressive accumulation of atherosclerotic plaques in blood vessels, leading to potentially fatal vascular events [2]. The treatment of ASCVD generally includes lifestyle interventions, pharmacological therapies, and, in some cases, invasive procedures [3]. Despite advances in treatment strategies, a significant proportion of patients do not reach the recommended targets for low-density lipoprotein cholesterol (c-LDL) [4,5].

In recent years, considerable attention has been given to the development of new pharmacological agents to reduce the risk of ASCVD attributable to lipid components. The introduction of PCSK9 inhibitors has shown excellent results in terms of reducing c-LDL levels and vascular events. Now, the arrival of inclisiran further improves the therapeutic arsenal [6,7]. Inclisiran is a first-in-class interfering RNA (siRNA) therapy that inhibits proprotein convertase subtilisin/kexin type 9 (PCSK9) [8]. Extensive randomized clinical trials, particularly the ORION studies, have demonstrated that inclisiran effectively and safely reduces c-LDL levels by up to 52% in patients with established ASCVD or those at high risk of developing cardiovascular disease [8,9,10,11]. This substantial and sustained reduction in c-LDL levels is associated with a decrease in the incidence of major adverse cardiovascular events, including myocardial infarctions and strokes [10]. The efficacy of inclisiran in reducing c-LDL and its favorable pharmacokinetic profile—requiring administration only twice a year—makes it a promising therapeutic option for patients with ASCVD, especially those at high or very high cardiovascular risk. Moreover, the drug’s ability to achieve c-LDL reduction with more convenient administration may help improve patient adherence, thereby increasing the likelihood of achieving long-term lipid management goals [11]. Despite the encouraging results obtained in the randomized clinical trials, real-world results often differ, among other reasons, due to differences in patient characteristics between clinical trials and routine practice [12,13].

This study aims to systematically review and analyze the evidence on the efficacy of inclisiran in reducing c-LDL levels and in preventing adverse cardiovascular events in patients with ASCVD in real-life settings. By synthesizing data from multiple real-world cohorts, we seek to evaluate and compare the overall clinical impact of inclisiran as a key component in the management of ASCVD in the real world with the results obtained in clinical trials [14,15].

## 2. Materials and Methods

This review has been conducted adhering to the guidelines of PRISMA (Preferred Reporting Items for Systematic Reviews and Meta-Analysis) [16], but was not registered. To find real-world evidence of inclisiran, an electronic search was conducted on 19 March 2025, in the MEDLINE database via the PubMed search engine without language restrictions using the following search terms. The first search term aims to find real-world studies, while the second term refers to the drug inclisiran per se:

(“Treatment Outcome”[MeSH] OR “Evidence-Based Medicine”[MeSH] OR “Retrospective Studies”[MeSH] OR “Time Factors”[MeSH] OR “real world” OR “real-world” OR “RWD” OR “RWE” OR “real life” OR “real patient” OR “real practice” OR “real clinical” OR “real population” OR “actual world” OR “actual life” OR “actual patient” OR “actual practice” OR “actual clinical” OR “actual population”)

AND

((ALN PCSsc[Title/Abstract]) OR (ALN 60212[Title/Abstract]) OR (PCSK9si KJX 839[Title/Abstract]) OR (Inclisiran[Title/Abstract]) OR (small interfering RNA[Title/Abstract]) OR (RNAi[Title/Abstract]) OR (SiRNA[Title/Abstract]) OR (RNA, Small Interfering[Title/Abstract])).

[17,18]

The inclusion terms were set as follows: (1) cohort studies, both prospective and retrospective, (2) in patients, (3) that were in real life, (4) in which the reduction in c-LDL was evaluated, and (5) that used the lipid-lowering drug inclisiran. The exclusion criteria were established to eliminate those works that do not strictly meet the previously mentioned inclusion criteria.

**Figure 1:**
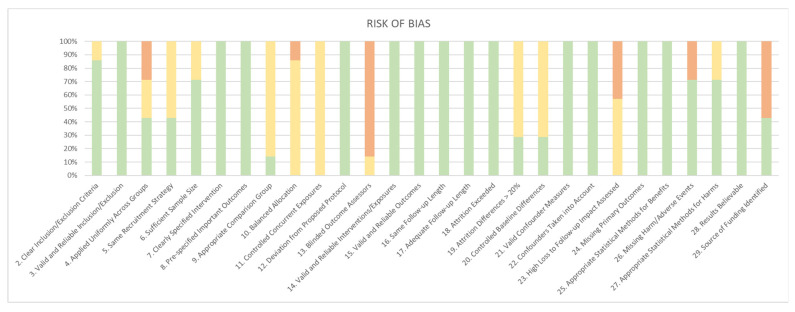
Results bias from RTI Item Bank.

To ensure the accuracy and transparency of the review, the results of this search were uploaded to an online program specialized in systematic reviews, Rayyan [19]. From the articles uploaded initially, those that were duplicated were detected by automated detection methods, whose results were reviewed and confirmed by the reviewing team. This team consisted of 3 people, 2 of whom (Rodrigo Alaíz A. and Miramontes González JP.) independently selected all remaining articles by reading their titles and abstracts, and selecting articles based on inclusion and exclusion criteria. Any discrepancies between the two article analysts were resolved by the third team member (Corral Gudino L.). The full text of articles that met all inclusion criteria were reviewed by both reviewers simultaneously, and if there were discrepancies, they were resolved by the third member, always following this method. The risk of bias of the finally selected articles was individually evaluated using a questionnaire of 29 questions called Item Bank for Assessment of Risk of Bias and Precision for Observational Studies of Interventions or Exposures (RTI Item Bank) [20]. The results of this analysis are presented in a figure (Figure 1) that summarizes them using various color schemes. A substantial risk of bias is indicated in red, a low risk in green, and an inability to identify the risk due to inadequate information or intermediate bias is indicated in yellow.

For the meta-analysis, data on mean reduction in low-density lipoprotein cholesterol (c-LDL) and its standard deviation (SD) directly reported in the included studies were used. In cases where the articles presented results in the form of medians, interquartile ranges (IQRs) or confidence intervals (CIs), these values were transformed into means and standard deviations using validated mathematical formulas for this purpose. These transformations allowed for the homogeneous inclusion of the data in the statistical analysis, ensuring comparability and robustness of the obtained results.

The meta-analysis was conducted using the Review Manager (RevMan) version 5.4 tool, developed by the Cochrane Collaboration. This software is widely recognized as a standard in conducting systematic reviews and meta-analyses, allowing efficient data management, statistical synthesis of results, and graphical representation through forest plots. The statistical methods recommended by Cochrane were employed to evaluate the combined effect. The methodological quality of the studies was previously assessed to guarantee the robustness and validity of the obtained results [21].

## 3. Results

**Figure 2:**
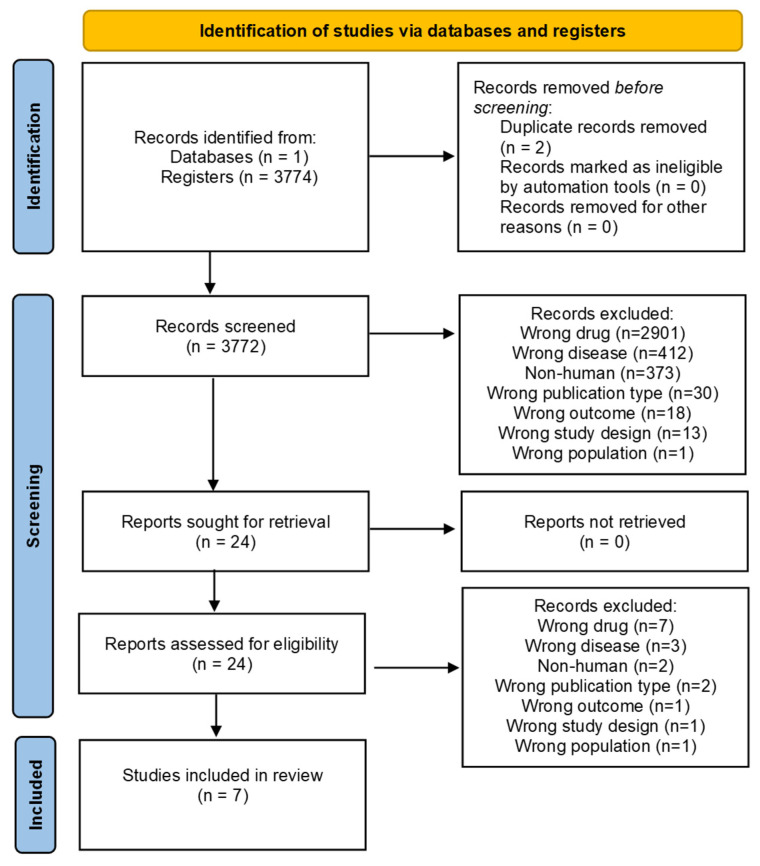
PRISMA Flow Chart.

After conducting the search, 3774 studies were initially obtained, of which 2 were identified through automated tools from Rayyan as duplicates and were confirmed as such by the reviewing team. The remaining 3772 articles were reviewed by two team members in parallel and with Blind On selected (Rodrigo Alaíz A. and Miramontes González JP.), reading the title and abstract and evaluating whether they met the inclusion and exclusion criteria. Those that met the criteria were marked as valid, and those that did not meet the inclusion criteria were marked as excluded, and in addition to being discarded, they were assigned a label categorizing the reason for their exclusion. Any discrepancies in this or the subsequent selection processes were evaluated by the third team member (*Corral Gudino* L.) and a consensus was reached unanimously. After that, 2901 articles discussing a different drug than the one being included were discarded and categorized as wrong drug, 412 that did not discuss atherosclerosis were categorized as wrong disease, 373 that did not have human participants were labeled as non-human, 30 that used neither prospective nor retrospective cohorts were categorized as wrong publication type, and 18 that did not measure a decrease in c-LDL levels were labeled as wrong outcome. With the 24 remaining articles, reading of the full-text articles was conducted to verify that they indeed met the criteria, following the same process as the previous screening. In total, 17 of them were discarded; 7 articles discussing a different drug than the one being included were discarded and categorized as wrong drug, 3 that did not discuss atherosclerosis were categorized as wrong disease, 2 that did not have human participants were labeled as non-human, 2 that used neither prospective nor retrospective cohorts were labeled as wrong publication type, and 1 that did not measure a decrease in c-LDL levels was categorized as wrong outcome, finally obtaining 7 articles [22,23,24,25,26,27,28]. The entire process is summarized, as can be seen in the PRISMA Flow Chart (Figure 2).

All of the selected studies provided valid and reliable information regarding the methodology, with an appropriate consideration of potential confounding biases, participant allocation, and analysis of the benefits obtained. However, some studies lacked information on adverse events [26], and in several cases, the source of funding was not specified [22,25,26]. Figure 1 provides an assessment of the methodological quality of the included studies based on key questions, following the protocol called Item Bank for Assessment of Risk of Bias and Precision for Observational Studies of Interventions or Exposures (RTI Item Bank) [20]. Each study is analyzed based on aspects related to design, validity, statistical analysis, and transparency, among others. The main differences in the studies refer to the handling of random allocation, participant follow-up, and bias control. The studies by Mulder (2023) [24] and Gargiulo (2025) [28] presented a robust prospective design, while the other included studies were retrospective, which may affect the ability to infer causality. Other differences include the duration of observation; the study by Wei (2024) [27] evaluates a period of 3 to 12 months, while the rest of the studies follow up for more than 2 months and 3 months (enough time for the drug to reach its efficacy).

The analysis of the sociodemographic characteristics is reflected in Table 1. Of the 1454 individuals from the 7 cohorts, 649 were women, which accounts for 44.64%. Two of them were prospective cohorts and the other five were retrospective. The clinical characteristics and results of the participants included in the studies were as follows. Table 1 summarizes the baseline characteristics of seven studies investigating the effect of inclisiran in real-world settings. The studies vary in design, geographical location, sample size, and the demographic characteristics of the participants, offering a diverse perspective on the use of this hypolipidemic agent.

**Figure 3:**
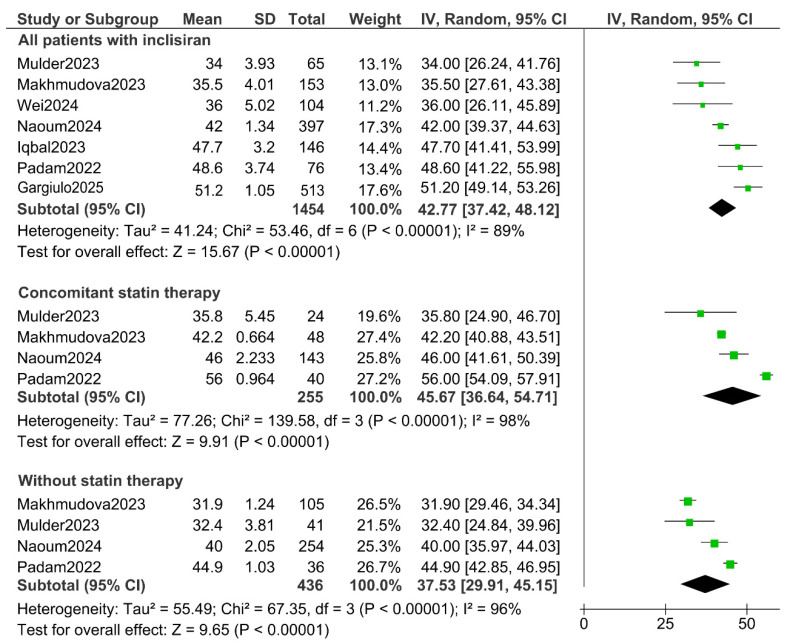
Overall forest plot results and subgroup analyses (concomitant statin therapy and monotherapy) [22,23,24,25,26,27,28].

The LDL reductions (Figure 3) vary from a maximum of 51.2% in the study by Gargiulo (2025) [28] (the study with the most patients (*n* = 513)), to a minimum of 34% in the study by Mulder (2023) [24]. These results have been analyzed based on the treatment being a monotherapy or concomitant with statins. In monotherapy, inclisiran demonstrated a reduction ranging from 31.9% [23] to 44.9% [22]. The results depending on the concomitant use of statins show a reduction ranging from 42.2% [23] to a maximum of 56% [22].

After conducting the meta-analysis, which included a total of seven studies with 1454 patients, it was observed that the use of inclisiran in real clinical practice conditions achieves an average reduction in low-density lipoprotein cholesterol of 42.77%. This effect was estimated with a 95% confidence interval (CI), ranging from 37.42% to 48.12%, as well as a reduction in those taking statins concomitantly of 45.67% (36.64–54.71%) compared to 37.53% (29.91–45.15) in those who did not take them (Figure 3).

There was considerable heterogeneity among the included studies. In the overall analysis of all of the patients treated with inclisiran (*n* = 1454), the I^2^ statistic reached 89%, with a τ^2^ of 41.24 and a significant Q test (χ^2^ = 53.46; *p* < 0.00001), indicating that the variability in effect sizes exceeded what would be expected by chance. The subgroup analyses did not attenuate this dispersion: in the patients receiving concomitant statins (*n* = 255), the I^2^ was 98% (τ^2^ = 77.26; χ^2^ = 139.58; *p* < 0.00001), whereas in those not on statins (*n* = 436), the I^2^ was 96% (τ^2^ = 55.49; χ^2^ = 67.35; *p* < 0.00001). These values confirm substantial heterogeneity across all of the strata analyzed, so a random-effects model was employed.

## 4. Discussion

The use of therapies targeting the PCSK9 protein began a new era of lipid-lowering treatment, enabling many patients to achieve LDL-C reductions in line with increasingly stringent guidelines. These therapies also provide an alternative for statin-intolerant patients. Inclisiran stands out for its convenient dosing regimen, which enhances therapeutic adherence, quality of life, and for its negligible adverse effects. The ORION clinical trials have demonstrated LDL-C reductions of up to 52%, which would allow a large number of patients to achieve therapeutic LDL levels. However, real-world studies have revealed notable discrepancies (Figure 3).

As shown, the analysis of the total patients (1454 individuals) shows an average reduction in the baseline c-LDL of 42.77% (CI: 37.42–54.71%), which is notably lower than the 52% reduction observed in controlled clinical trials (8,10). As we show in the results, it should be taken into account the use of the drug in monotherapy and with statins. In the ORION studies, inclisiran monotherapy resulted in an LDL-C reduction of 48.8%, whereas an analysis of the real-world data showed a reduction of 37.5% (29.9–45.2%), with the best result achieved by Padam (2022), with 48.6% [22]. When combined with statins, the LDL-C reduction improved to 45.7% (36.6–54.7%), although this still falls short of the 54.5% reduction seen in the ORION trials [29].

The impact of ezetimibe as an adjunct therapy should also be considered. Among the seven real-world studies analyzed, only the trials by Makhmudova (2023) [23] and Padam (2022) [22] reported data on ezetimibe therapy, with mixed results (7.8% further reduction versus no significant change). However, the sample size of patients receiving both ezetimibe and statins was limited (11 and 19 individuals, respectively). Of note, in the ORION studies, ezetimibe was not evaluated in isolation [9,11].

The results of randomized controlled trials (RCTs) usually differ to those obtained in real-world cohort studies due to key differences in design, population, and context. RCTs are conducted in strictly controlled conditions to minimize biases, with specific inclusion/exclusion criteria that select homogeneous patients. However, real-world studies include more diverse populations, better reflecting variations in comorbidities, adherence, and clinical practices [12,30]. Furthermore, in RCTs, the participants are highly supervised, which promotes an adherence close to 100%. In the real world, adherence can be much lower, affecting the observed effectiveness [31,32].

Another significant difference is outcome measurement; while RCTs usually use predefined and standardized endpoints, real-world studies rely on routine clinical registry data, which may be incomplete, disorganized, or inconsistent [32]. Additionally, observational studies are subject to biases such as selection (non-randomized patients) or confounding (uncontrolled factors influencing outcomes) [32,33,34]. Finally, RCTs and real-world studies have different objectives. While RCTs seek to establish causality, real-world studies focus on effectiveness in practical conditions, complementing information from RCTs with a more applicable approach to daily clinical practice and can yield great value to understand how the drug behaves in more heterogeneous populations [35,36]. All of these differences underscore the importance of using both sources of evidence complementarily, as RCTs provide robust estimates of causality, and real-world studies help assess the applicability in everyday clinical contexts [30].

The 9% gap between the mean LDL-C reduction reported in the ORION trials (52%) and the effect we observed under real-world conditions (42.77%) warrants careful interpretation. First, a “law of diminishing returns” applies to the baseline LDL-C: for each additional mmol/L at baseline, the absolute percentage drop tends to plateau [12,30]. Our data reproduce this pattern: the studies with a baseline LDL-C < 3 mmol/L [28] achieved >50% reductions, whereas the cohorts starting > 3.5 mmol/L fell well below that mark.

Second, even with a twice-yearly regimen, adherence in routine care rarely approaches 100%. Logistical hurdles—delays in scheduling the second injection and hospital pharmacy dispensing issues—and the patient’s perception of good health after the first dose frequently led to late administrations [31], reducing the cumulative exposure and thus the 3- to 6-month efficacy. Finally, concomitant comorbidities (metabolic syndrome, obesity, chronic inflammation) and therapeutic inertia in up-titrating statins or ezetimibe can blunt the lipid response. Recognizing these factors is essential for setting realistic expectations, reinforcing on-schedule adherence, and selecting the most appropriate candidates for inclisiran in everyday practice.

Furthermore, and as a noteworthy fact, it should be taken into account that the baseline c-LDL levels in some of these controlled studies such as ORION 10 and ORION 11 were already low, at around 2.6 mmol/L of LDL, while the average of the analyzed real-world studies is around 20% higher, at around 3.15 mmol/L, which the drug then has to reduce [29]. This is in line with the results obtained in this systematic review. The patients in the cohort from Gargiulo (2025) [28], who started from the lowest baseline levels of c-LDL out of the seven studies (2.7 mmol/L), achieved one of the highest reductions, which was 51.2%; meanwhile, the patients from Mulder (2023) [24], who started with higher levels of c-LDL (3.7 mmol/L), only achieved a reduction of 34% [24,28].

The time elapsed until the analysis of the published data emerges as a pivotal aspect, potentially explaining why the 50% reduction target may not always be achieved. For instance, in the ORION 11 trial, the final outcomes were assessed at 18 months. However, significant reductions of 40% were observed as early as 90 days. This early response period at 90 days warrants consideration, particularly in real-world settings where substantial inter-individual variability in response between 3 and 9 and 12 months is notable. Notably, as evidenced in German registries, outcomes often exhibit a preferable response at 90 days, underscoring the relevance of including this timeframe in our discussion.

Another way to analyze the efficacy of the drug is to evaluate the number of patients achieving a desired cLDL reduction, that is, in therapeutic targets according to ESC, in high-risk (<70 mg/dL) and very high-risk (<55 mg/dL) patients [37]. In the analyzed studies, only two studies measured this parameter. Iqbal (2024) [25]., with a 3-month follow-up of 146 patients, showed a mean cLDL reduction of 47.7%, with the percentages of patients in range being 16% and 32% in very high and high risk groups, respectively. Gargiulo (2025) [28], with the same follow-up duration, showed a mean cLDL reduction of 51.2% with the percentages of patients in range being 57% and 69% in very high and high risk groups, respectively [25,28].

On the other hand, the study by Naoum (2024) [26] reported a median reduction in cLDL in a retrospective cohort, more than 2 months after the first administration, in 397 patients, stating that a 42% reduction occurred. This work highlights that a greater LDL reduction is achieved in individuals taking other lipid-lowering treatments concomitantly than in those who do not, at 46% (*n* = 143) and 40% (*n* = 254), respectively, which is an expected fact [26].

This study has several limitations that should be considered when interpreting the results. First, the inclusion of data converted into means ± standard deviations may introduce some uncertainty, because these transformations rely on statistical assumptions that may not fully capture the original data distribution. Second, the high statistical heterogeneity observed (I^2^ ≥ 89% in all analyses) indicates substantial clinical and methodological differences among the included studies. Potential sources include variations in the inclisiran dosing regimen, follow-up duration, baseline LDL levels, concomitant statin therapy, laboratory analytical methods, and patient selection criteria. Although the magnitude of the pooled effect remains clinically relevant, the wide confidence intervals and inconsistency across studies limit the direct generalizability of the findings. Meta-regression and sensitivity analyses are needed to systematically explore these sources of variability. In addition, the observational nature of real-world studies may be subject to uncontrolled biases, such as confounding. Although the RTI Item Bank indicated an overall low-to-moderate risk of bias, the non-randomized nature of the included studies poses important threats to internal validity. Inclisiran is typically prescribed for patients with particularly challenging lipid profiles or statin intolerance; such confounding by indication could underestimate or overestimate the true effect if the same factors driving prescription also influence response [32,34].

In addition, with only seven cohorts available, we lacked power for formal tests such as Egger’s regression or trim-and-fill, yet the size asymmetry—smaller studies tending to report greater reductions—hints at a possible publication bias toward favorable outcomes. Future incorporation of administrative databases and national registries should reduce this asymmetry and allow for adequately powered funnel plot analyses. Until then, clinicians should interpret the magnitude of effect cautiously and base therapeutic decisions more on residual cardiovascular risk than on an isolated percentage decrease. Finally, the limited number of included studies and their relatively small sample sizes restrict the statistical power of the meta-analysis, underscoring the need for future investigations with broader scope, larger populations, and greater methodological rigor to confirm these results.

## 5. Conclusions

Inclisiran has been shown, in real-world trials, to be a potent lipid-lowering agent, with a more convenient dosing regimen than other drugs targeting the PCSK9 protein, and great safety concerning side effects. Furthermore, this work shows its effectiveness when combined with other lipid-lowering agents, particularly statins. A discrepancy with controlled trials has been observed, which may stem from study designs and the characteristics of the patients included in each, especially the baseline LDL-c levels in the different studied cohorts. This difference may be clarified with the publication of more real-world studies and a longer follow-up in them.

## Figures and Tables

**Table 1:** Sociodemographic and baseline characteristics of the selected studies. (* the value is a standard deviation, ** the value is an interquartile range).

Study	Design	Country	Population	Follow-Up
Padam (2022) [22]	Retrospective observational	UK	76	2 months
Makhmudova (2023) [23]	Retrospective observational	Germany	153	3 months
Mulder (2023) [24]	Prospective observational	Netherlands	65	3 months
Iqbal (2024) [25]	Retrospective observational	UAE	146	3 months
Naoum (2024) [26]	Retrospective observational	Israel	397	>2 months
Wei (2024) [27]	Retrospective observational	USA	104	3–12 months
Gargiulo (2025) [28]	Prospective observational	Italy	513	3 months
STUDY	MEAN AGE (SD */IQR **)	FEMALE (%)	c-LDL BASELINE (mmol/L) (SD */IQR **)	
Padam (2022) [22]	64 (10.3 *)	28 (35%)	3.5 (1.1 *)	
Makhmudova (2023) [23]	63 (55.70 **)	66 (43.1%)	3.6 (2.4 **)	
Mulder (2023) [24]	63 (54.68 **)	36 (55%)	3.7 (2.5 **)	
Iqbal (2024) [25]	54.8 (12.12 **)	64 (43.8%)	3.0 (1.8 **)	
Naoum (2024) [26]	66 (11 *)	226 (56.9%)	-	
Wei (2024) [27]	67.5 (11.1 **)	70 (67.3%)	3.1 (1.7 *)	
Gargiulo (2025) [28]	63	159 (31%)	2.7 (1.8 **)	

## Data Availability

Not applicable.
